# Development and Preliminary Evaluation of an OMP16-Targeting Trivalent Nanobody-HRP-Based cELISA for Serological Detection of Bovine Brucellosis

**DOI:** 10.3390/ani16111707

**Published:** 2026-06-03

**Authors:** Gaowa Wudong, Qing Lu, Yunyi Zhai, Ye Yuan, Xiaofang Liu, Yuanhao Yang, Lu Zhang, Yaping Jin, Dong Zhou, Aihua Wang

**Affiliations:** 1 College of Veterinary Medicine, Northwest A&F University, Xianyang 712100, China; wudonggaowaa@163.com (G.W.);; 2 Key Laboratory of Animal Biotechnology of the Ministry of Agriculture, Northwest A&F University, Xianyang 712100, China

**Keywords:** *Brucella*, OMP16, 3Nbs-HRP, cELISA

## Abstract

Brucellosis is a globally prevalent zoonotic disease that imposes considerable economic burdens on the livestock industry and remains a significant threat to public health. Although lipopolysaccharide (LPS)-based serological assays are widely used in routine diagnosis, their inherent limitations—particularly cross-reactivity with other Gram-negative bacteria—underscore the need for the development of diagnostic approaches based on non-LPS antigens. In this study, we developed a trivalent nanobody–horseradish peroxidase (3Nbs-HRP) fusion protein targeting *Brucella* OMP16 and established a cELISA for the serological detection of bovine brucellosis. The diagnostic performance of the assay was assessed using 204 *Brucella* antibody-negative and 123 *Brucella* antibody-positive bovine serum samples. ROC curve analysis yielded a sensitivity of 87.7% and a specificity of 89.4%, with no significant cross-reactivity observed. By employing a recombinant antigen and a 3Nbs-HRP probe, this assay enhances biosafety and demonstrates strong potential for standardization and large-scale application, serving as a complementary tool to conventional LPS-based assays for the surveillance, diagnosis, and control of bovine brucellosis.

## Simple Summary

Brucellosis is an infectious disease that affects animals and can be transmitted to humans, leading to major economic losses in livestock production and posing risks to public health. Reliable diagnosis is essential for controlling its spread; however, many current tests may give false results because they react with bacteria other than *Brucella*. In this study, we developed a novel blood test based on a conserved protein from *Brucella* and a specially engineered small antibody. To improve detection performance, three identical antibody units were combined into a single molecule and directly linked to a signal-generating enzyme, simplifying the testing process. The method was evaluated using cattle serum samples and showed good accuracy without cross-reaction. This approach provides a potentially safer, more specific, and easier-to-use diagnostic tool and may serve as a valuable complement to existing methods for the control of bovine brucellosis.

## 1. Introduction

Brucellosis is a globally prevalent zoonotic disease caused by bacteria of the genus *Brucella*, posing a significant threat to both public health and the livestock industry [1]. In livestock, the disease primarily leads to abortion and infertility, whereas in humans, it may result in acute or chronic debilitating disease, often accompanied by complications such as arthritis, endocarditis, and meningitis [2,3]. Despite long-standing control efforts, the true global burden of brucellosis remains underestimated. Recent modeling studies have estimated approximately 2.1 million new cases annually, with the highest incidence reported in Africa and Asia [4]. In China, brucellosis is classified as a class II notifiable animal disease, and infection rates in livestock have increased in recent years [5]. The disease continues to cause substantial economic losses worldwide and presents ongoing challenges for surveillance, diagnosis, and control [6,7,8].

Laboratory diagnosis of brucellosis primarily includes bacterial isolation, molecular methods such as PCR, and serological assays. In endemic regions, serological testing remains the primary diagnostic approach due to its relatively high sensitivity and specificity, as well as its cost-effectiveness [9,10,11]. Among these methods, ELISA has become an important tool for serological diagnosis because of its reliability, ease of operation, and suitability for large-scale screening. However, most ELISAs are based primarily on smooth lipopolysaccharide (S-LPS), which may cross-react with other Gram-negative bacteria, thereby reducing diagnostic specificity [12,13].

To overcome the limitations of LPS-based diagnostic methods, increasing attention has been directed towards *Brucella* protein antigens. Outer membrane proteins (OMPs) are important immunogenic and protective antigens, closely associated with bacterial virulence [14,15], and thus play a critical role in *Brucella* diagnostics. Common OMPs, such as BP26 and OMP25, show good diagnostic performance in some *Brucella* species. However, BP26 exhibits species- and strain-dependent variability, which may limit its ability to detect all animals infected with *Brucella* [16]. Similarly, OMP25 shows sequence variation among different *Brucella* strains, which impacts its application in broad screening [17,18].

In contrast, OMP16 is a highly conserved outer membrane protein [19]. It not only maintains the integrity of the outer membrane, which is crucial for bacterial survival [14,20], but also plays a key role in *Brucella* virulence, both in vitro and in vivo [14]. Studies have shown that OMP16 is critically involved in the pathogenesis of *Brucella* infections and exhibits high immunogenicity [21]. Golchin et al. [12] developed an indirect ELISA using OMP16, showing 100% sensitivity and 95.0–100% specificity in human serum samples. The assay showed good agreement with the Rose Bengal plate test and a commercial ELISA kit. These findings further support the significant potential of OMP16 as a candidate for *Brucella* diagnosis and vaccine development.

Despite these advantages, highly specific and stable serological assays based on OMP16 remain scarce. Nanobodies (Nbs), derived from camelid heavy-chain-only antibodies, exhibit several advantageous properties, including small size, high stability, ease of genetic manipulation, and strong binding affinity [22,23,24]. These characteristics make them attractive tools for infectious disease diagnosis.

In this context, the goal of this study is to develop a novel non-LPS-based diagnostic method, specifically a cELISA platform utilizing OMP16-specific nanobody technology. By employing a trivalent nanobody-HRP fusion probe, we aim to enhance the specificity of *Brucella* serological testing. This approach is designed to overcome the limitations of existing LPS-based assays, particularly in terms of cross-reactivity, providing a simpler and more efficient diagnostic tool. The platform holds the potential to play a significant role in *Brucella* surveillance and control, serving as a valuable complement to current diagnostic strategies.

## 2. Materials and Methods

### 2.1. Experimental Materials

The recombinant OMP25 protein, GST protein, pEGFP-N1-RANbody vector, pGEX-4T-1-OMP16 plasmid, *Escherichia coli* TG1 strain, and all non-*Brucella* pathogen-positive sera used for specificity testing were maintained in our laboratory. The commercial *Brucella* cELISA kit and the standard tube agglutination test (SAT) kit were purchased from Qingdao Lijian Bio-Tech Co., Ltd. (Qingdao, China). In addition, 204 *Brucella* antibody-negative and 123 antibody-positive bovine serum samples were included. Classification of the serum samples as antibody-positive or -negative was based on concordant results from multiple diagnostic approaches, including the commercial cELISA, SAT, and PCR analysis. For PCR testing, rectal, nasal, or vaginal swabs were collected from the corresponding animals, and a sample was considered PCR-positive if any of the swabs tested positive.

### 2.2. Expression and Purification of OMP16 Protein

*E. coli* BL21(DE3) competent cells (Weidi Biotechnology Co., Ltd., Shanghai, China) were transformed with the pGEX-4T-1-OMP16 plasmid and cultured at 37 °C until reaching the mid-logarithmic phase. Protein expression was induced with 0.5 mM IPTG and maintained at 22 °C for 8 h [25]. Cells were collected by centrifugation at 10,000× *g* for 10 min, followed by cell lysis. The supernatant was then harvested and the recombinant GST-OMP16 fusion protein was purified using glutathione Sepharose affinity resin (Smart-Lifesciences, Changzhou, China) [25]. Protein purity was analyzed by SDS-PAGE. The purified protein was subsequently dialyzed, concentrated, and stored for further use.

### 2.3. Construction of an OMP16-Specific VHH Phage Display Library

Purified GST-OMP16 (3 mL, 2 mg/mL) was mixed with an equal volume of complete Freund’s adjuvant (Sigma-Aldrich, St. Louis, MO, USA) to form an emulsion, which was then administered subcutaneously to a camel. The animal was maintained under standard husbandry conditions with free access to food and water and managed in accordance with relevant animal care and use guidelines. Booster immunizations were given every two weeks using 6 mg of GST-OMP16 emulsified with an equal volume of incomplete Freund’s adjuvant (Sigma-Aldrich, St. Louis, MO, USA). Six days after the fifth booster immunization, peripheral blood was collected, and serum was separated to determine anti-GST-OMP16 antibody titers. Antibody titers were measured by iELISA using GST-OMP16-coated plates (NEST Biotechnology Co., Ltd., Wuxi, China) at a coating concentration of 2 μg/mL, with serially diluted sera, pre-immune serum as a negative control, and HRP-conjugated rabbit anti-camel IgG (Solarbio Science & Technology Co., Ltd., Beijing, China) as the secondary antibody.

The VHH phage display library was constructed as previously described [26]. Briefly, total RNA was extracted from peripheral blood lymphocytes, followed by cDNA synthesis. VHH fragments were amplified by nested PCR, cloned into a phagemid vector, and transformed into *Escherichia coli* TG1 to generate the primary library. The library size was estimated by counting colonies on selective plates. To assess library quality, 48 colonies were randomly selected for colony PCR to verify the presence of VHH inserts, with a previously confirmed VHH-containing clone and an empty phagemid vector used as positive and negative controls, respectively, and the insertion rate was calculated. PCR-positive clones were further subjected to Sanger sequencing to evaluate sequence diversity.

### 2.4. Screening and Characterization of OMP16-Specific Nanobodies

Phage biopanning against GST-OMP16 was performed for three rounds as previously described [26] to enrich OMP16-specific phage particles. Phage rescue was performed after each round. After the third round of selection, ninety-six colonies were randomly selected, cultured, and induced for soluble expression of VHHs. Soluble VHH extracts were prepared and screened by iELISA to assess their binding activity toward GST-OMP16. Positive clones were sequenced and subsequently grouped based on variations in the complementarity-determining region 3 (CDR3) amino acid sequences.

To assess specificity, GST protein, *Brucella* OMP25, and GST-OMP16 were each coated onto 96-well plates at 4 μg/mL. After incubation with soluble VHH extracts, bound nanobodies were detected using a mouse anti-HA monoclonal antibody (TransGen Biotech Co., Ltd., Beijing, China) followed by HRP-conjugated goat anti-mouse IgG (Wuhan Sanying Biotechnology Co., Ltd., Wuhan, China). The reaction was developed with TMB (Solarbio Science & Technology Co., Ltd., Beijing, China) for 15 min, stopped with 2 M H_2_SO_4_, and absorbance was measured at 450 nm using a microplate reader (Bio-Rad Laboratories, Inc., Hercules, CA, USA).

To compare the relative binding activities among OMP16-specific nanobodies, GST-OMP16 was coated onto ELISA plates, and soluble VHH extracts were serially diluted at 1:5, 1:10, 1:100, and 1:1000 before being added to the coated plates. Binding activities were then analyzed by iELISA.

### 2.5. Construction, Expression, and Functional Characterization of 3Nbs-HRP Fusion Proteins

To construct homotrimeric nanobody-HRP fusion proteins, three identical VHH sequences corresponding to each nanobody were tandemly assembled by overlap extension PCR. The individual VHH domains were connected via flexible (Gly4Ser) linkers to ensure proper folding and functional independence of each domain. The resulting trimeric VHH fragments were cloned into the pEGFP-N1-RANbody vector by restriction digestion and ligation, generating recombinant expression plasmids designated as pEGFP-3Nbs-HRP. All constructs were confirmed by Sanger DNA sequencing.

HEK293T cells were seeded into 6-well plates and cultured under standard conditions (37 °C, 5% CO_2_) until reaching 70–80% confluency. Sequence-verified pEGFP-3Nbs-HRP plasmids were individually transfected into HEK293T cells. At 72 h post-transfection, supernatants from HEK293T cell cultures were collected and used for subsequent analyses.

To evaluate intracellular expression, immunofluorescence staining was performed at 48 h post-transfection. Cells were fixed with pre-chilled 70% ethanol, washed with PBST, and blocked with 1% BSA (Solarbio Science & Technology Co., Ltd., Beijing, China) at 37 °C for 1 h. Cells were then incubated with a mouse anti-HA monoclonal antibody, followed by Alexa Fluor 488-conjugated goat anti-mouse IgG (Beyotime Biotechnology, Shanghai, China). Fluorescence signals were observed and imaged using a fluorescence microscope (Leica Microsystems, Wetzlar, Germany).

The binding activities of the homotrimeric 3Nbs-HRP fusion proteins were assessed by iELISA. Recombinant GST-OMP16 protein was coated onto 96-well plates, followed by blocking and washing. Culture supernatants were added at serial dilutions of 1:10, 1:100, 1:1000, 1:10,000, and 1:100,000 and incubated for 1 h. Color development was initiated by adding TMB substrate after the washing step. The enzymatic reaction was subsequently terminated with 2 M H_2_SO_4_, and the optical density was determined at 450 nm.

### 2.6. Development of 3Nbs-HRP-Based cELISAs

A cELISA based on 3Nbs-HRP fusion proteins was established for the rapid detection of antibodies against *Brucella* OMP16. The assay conditions were optimized by checkerboard titration, in which GST-OMP16 antigen was coated at concentrations ranging from 0.1 to 1 μg/mL and combined with serial dilutions of 3Nbs-HRP. Based on the OD450 values obtained for each combination, the antigen coating concentration and 3Nbs-HRP working dilution that produced an OD450 value close to 1.0 with stable signal intensity were selected for subsequent experiments.

To determine the optimal serum dilution, four *Brucella* antibody-positive and four negative sera were tested under the optimized coating conditions. ELISA plates were coated with the selected concentration of GST-OMP16, blocked with 3% gelatin (Solarbio Science & Technology Co., Ltd., Beijing, China), and washed with PBST. Serum samples diluted at 1:5, 1:10, 1:20, and 1:40 were mixed with 3Nbs-HRP and added to the wells (100 μL per well) for competitive binding. The optimal serum dilution was determined by comparing positive and negative sera across different dilutions in terms of OD values, inhibition efficiency, and P/N ratios. The dilution that provided effective discrimination between positive and negative samples, with stable signals and a relatively low average P/N ratio, was selected as the working dilution. To establish the diagnostic cutoff value, 204 *Brucella* antibody-negative and 123 positive serum samples were analyzed using the optimized 3Nbs-HRP-based cELISA. Percentage inhibition (PI) was defined as follows:

PI (%) = 100 × [1 − (OD450 of sample/OD450 of negative control)].

Receiver operating characteristic (ROC) curve analysis was performed using PI values as the test variable and the known serological status as the reference standard. The optimal cutoff value was determined based on the maximum Youden index (sensitivity + specificity − 1). Sensitivity and specificity were subsequently calculated at this cutoff using the standard definitions based on true positive, true negative, false positive, and false negative results.

### 2.7. Performance Evaluation of the 3Nb1A-cELISA

The analytical sensitivity of the 3Nb1A-cELISA was evaluated using four *Brucella* antibody-positive serum samples. Each sample was diluted to 1:5, 1:10, 1:20, 1:40, 1:80, and 1:160 and tested under optimized assay conditions. The limit of detection (LOD) was defined as the highest serum dilution that remained positive based on the established cutoff value.

The analytical specificity of the assay was assessed using *Brucella* antibody-positive sera and sera positive for *Yersinia enterocolitica* O:9, *Escherichia coli* K99, *E. coli* F17, *Salmonella*, *Mycobacterium bovis*, and foot-and-mouth disease virus (FMDV). For each non-*Brucella* pathogen, one positive serum sample was included. Cross-reactivity was evaluated by determining whether sera positive for non-*Brucella* pathogens produced PI values exceeding the cutoff value. Given the limited sample size, this analysis represents an initial evaluation of specificity. Assay repeatability was evaluated using a panel of three *Brucella* antibody-positive and three antibody-negative serum samples. For intra-assay variability, each sample was tested in triplicate within the same plate. For inter-assay variability, the same samples were tested in triplicate on three independent plates. The coefficient of variation (CV) was calculated to assess assay precision.

### 2.8. Comparison and Agreement Analysis of 3Nb1A-cELISA with SAT and a Commercial ELISA Kit

To evaluate the diagnostic agreement of the 3Nb1A-cELISA with established methods, a total of 288 field serum samples were collected from a dairy farm in Lingwu City, Ningxia Hui Autonomous Region, China. All samples were independently tested using the 3Nb1A-cELISA, the SAT, and a commercial ELISA kit according to their respective standard operating procedures. The positive percent agreement (PPA), negative percent agreement (NPA), and overall percent agreement (OPA) were calculated to assess diagnostic consistency. 

In addition, Cohen’s kappa coefficient (κ) was determined to evaluate the level of agreement between the assays, while McNemar’s test was used to determine whether the discordant classifications between paired methods differed significantly. Note that these field serum samples were used solely for the agreement analysis, and their infection status was not confirmed by gold-standard methods such as bacterial culture or PCR.

## 3. Results

### 3.1. Expression and Purification of Recombinant OMP16

**Figure 1:**
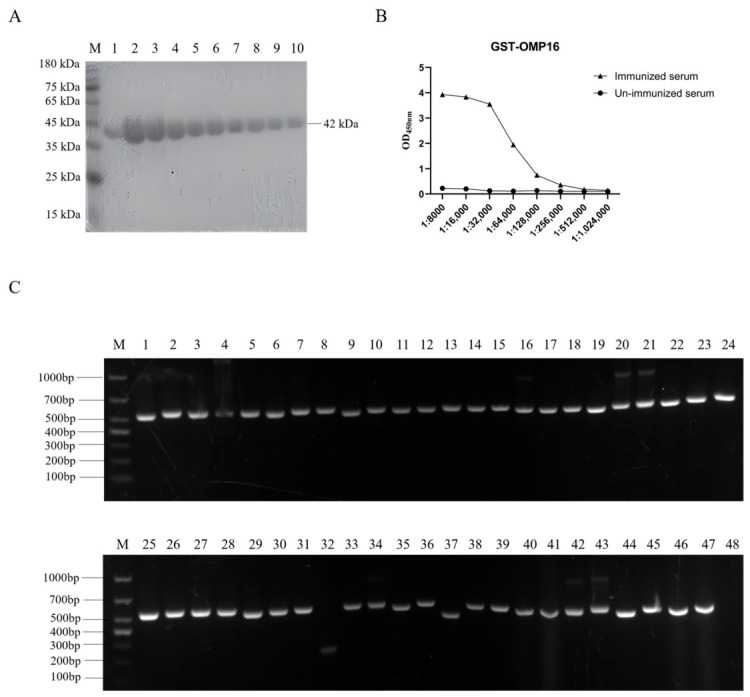
Expression of GST-OMP16 and construction of the OMP16-specific VHH library. (**A**) SDS-PAGE analysis of purified recombinant GST-OMP16. (**B**) Serum antibody titers against GST-OMP16 measured by iELISA. (**C**) Colony PCR analysis demonstrating the insertion rate of the VHH library. M, DL1000 DNA marker.

Expression of the recombinant GST-OMP16 fusion protein was analyzed by SDS-PAGE, revealing that the protein was predominantly expressed in the soluble fraction. The fusion protein was purified by glutathione Sepharose affinity chromatography. SDS-PAGE analysis of the eluted fractions confirmed the successful purification, showing a distinct band at approximately 42 kDa, consistent with the predicted molecular weight of GST-OMP16 (Figure 1A). The concentration of the purified fusion protein was determined using a BCA assay and adjusted to 2 mg/mL for camel immunization.

### 3.2. Construction and Characterization of an OMP16-Specific Phage Display VHH Library

The immune response in the camel was first evaluated by iELISA using plates coated with GST-OMP16. The serum antibody titer against GST-OMP16 reached 1:128,000 (Figure 1B), indicating a strong humoral immune response. PBMCs were isolated from the immunized camel’s blood, followed by extraction of total RNA and synthesis of cDNA. The resulting cDNA served as a template for nested PCR amplification of VHH fragments, which were subsequently ligated into a phagemid vector to construct an OMP16-specific VHH phage display library. The library size was approximately 8.3 × 10^8^ PFU/mL.

To assess the quality of the constructed library, 48 randomly selected clones were subjected to colony PCR, yielding a positive insertion rate of 95.83% (Figure 1C). Sequencing analysis of positive clones demonstrated substantial diversity within the OMP16-specific VHH library.

### 3.3. Isolation and Identification of OMP16-Specific Nanobodies

**Figure 2:**
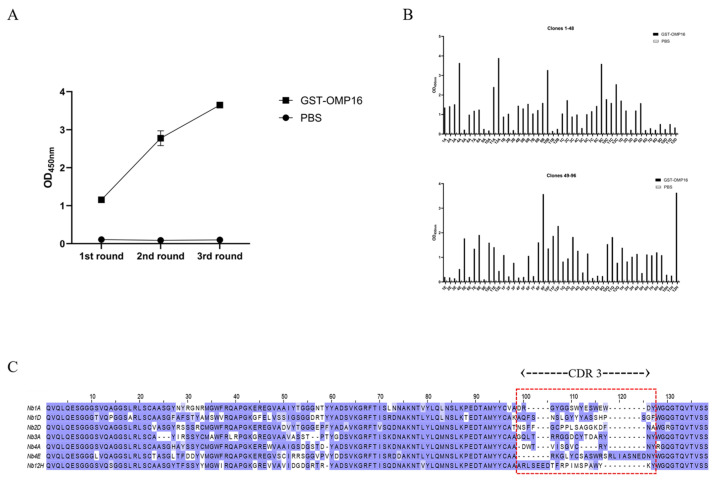
Screening of OMP16-specific nanobodies. (**A**) Enrichment of phages binding to GST-OMP16 during biopanning. (**B**) iELISA screening of candidate clones for binding to GST-OMP16. (**C**) Amino acid sequence alignment of seven OMP16-specific nanobodies. The CDR3 regions of the nanobodies are highlighted by the red dashed box and arrows.

Three rounds of biopanning were carried out using GST-OMP16 as the target antigen. Progressive enrichment of phage particles specific to GST-OMP16 was observed over successive selection rounds (Table 1 and Figure 2A), indicating effective enrichment of GST-OMP16-specific phages. Following the third round of selection, 96 individual clones were randomly selected and subjected to iELISA to evaluate their binding activity toward GST-OMP16. Among these, 64 clones demonstrated specific binding to GST-OMP16 (Figure 2B). Sequencing analysis of the positive clones identified seven unique OMP16-specific nanobodies based on variations in their CDR3 amino acid sequences (Figure 2C).

**Figure 3:**
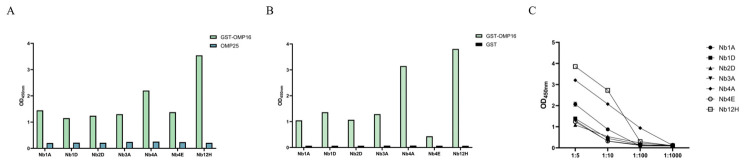
Specificity and binding activity of OMP16-specific nanobodies. (**A**) Cross-reactivity analysis against recombinant OMP25. (**B**) Cross-reactivity analysis against GST (pGEX-4T-1-derived tag protein). (**C**) Binding activity of nanobodies at serial dilutions as determined by iELISA.

The specificity of the selected nanobodies was further assessed using *Brucella* OMP25 (a recombinant protein) and GST (a tag protein derived from the pGEX-4T-1 expression system) as control antigens. ELISA analysis showed that all seven nanobodies specifically bound to GST-OMP16, with no detectable cross-reactivity to OMP25 (Figure 3A) or GST (Figure 3B).

To further compare their relative binding activities, crude nanobody extracts were serially diluted (1:5, 1:10, 1:100, and 1:1000) and analyzed by iELISA. Nb1A, Nb4A, and Nb12H consistently exhibited stronger binding signals across the dilution series, indicating higher apparent binding activity (Figure 3C). These three nanobodies were therefore selected for subsequent trivalent engineering and development of the cELISA.

### 3.4. Expression and Characterization of Trivalent Nanobody-HRP Fusion Proteins

**Figure 4:**
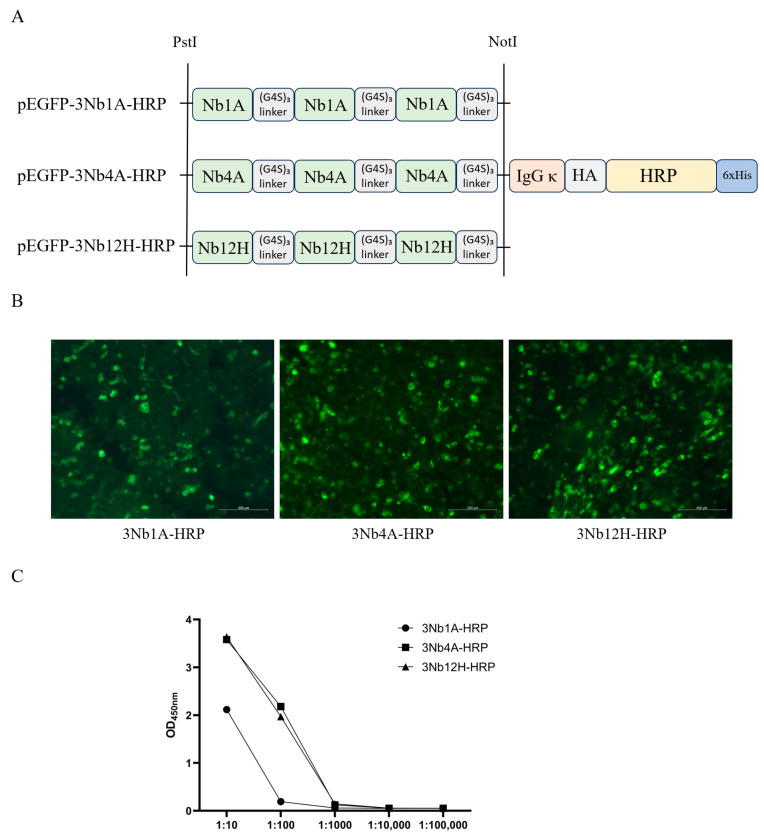
Construction and characterization of 3Nbs-HRP fusion proteins. (**A**) Schematic representation of the recombinant pEGFP-3Nbs-HRP plasmid. (**B**) IFA detection of 3Nbs-HRP fusion proteins. (**C**) iELISA analysis of the relative binding activity of 3Nbs-HRP fusion proteins.

To generate homotrimeric nanobody-HRP fusion proteins, three identical copies of Nb1A, Nb4A, and Nb12H were assembled in tandem and cloned into the pEGFP-N1-RANbody vector, generating pEGFP-3Nb1A-HRP, pEGFP-3Nb4A-HRP, and pEGFP-3Nb12H-HRP, respectively (Figure 4A). Transient transfection of HEK293T cells with the recombinant plasmids resulted in the expression of 3Nb1A-HRP, 3Nb4A-HRP, and 3Nb12H-HRP, as confirmed by immunofluorescence assay (IFA) at 48 h post-transfection (Figure 4B).

The relative binding activity of the expressed fusion proteins was subsequently evaluated by iELISA. All three fusion proteins exhibited measurable binding signals, with detectable binding activity at dilutions up to 1:100 (Figure 4C), indicating successful expression and retention of antigen-binding activity following trivalent engineering.

### 3.5. Development and Optimization of Three OMP16-Specific cELISAs

Checkerboard titration revealed that the optimal coating concentration of GST-OMP16 was 0.8 μg/mL for the 3Nb1A-cELISA and 1 μg/mL for both the 3Nb4A-cELISA and 3Nb12H-cELISA. Under these conditions, the optimal working dilutions of 3Nb1A-HRP, 3Nb4A-HRP, and 3Nb12H-HRP were 1:250, 1:80, and 1:100, respectively (Supplementary Tables S1–S3).

To determine the optimal serum dilution, four *Brucella* antibody-positive and four *Brucella* antibody-negative sera were tested at serial dilutions using the three cELISAs. A serum dilution of 1:5 was identified as the optimal dilution for all three assays and was therefore selected as the working serum dilution (Supplementary Table S4).

### 3.6. ROC Analysis and Cutoff Determination for Three cELISAs

**Figure 5:**
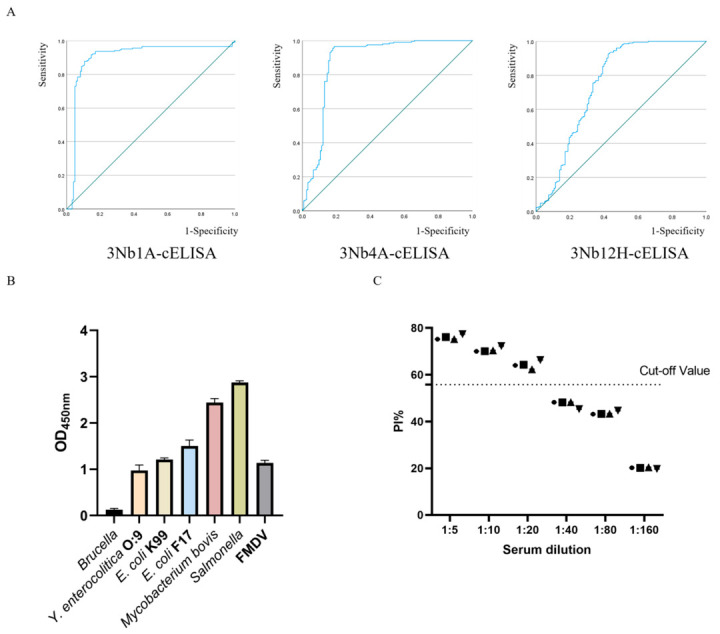
Diagnostic and analytical performance of the developed cELISAs. (**A**) ROC curve analysis. (**B**) Analytical specificity of the 3Nb1A-cELISA. (**C**) Analytical sensitivity and LOD.

We evaluated the diagnostic performance of three cELISAs—3Nb1A-cELISA, 3Nb4A-cELISA, and 3Nb12H-cELISA—using 123 *Brucella* antibody-positive and 204 *Brucella* antibody-negative bovine serum samples. ROC curves were constructed (Figure 5A), and the optimal cutoff values for each assay were determined based on the maximum Youden index (Table 2).

For the 3Nb1A-cELISA, the optimal cutoff was 55.78% PI, with a sensitivity of 87.7% and specificity of 89.4%. The 95% CI for the AUC was [0.858, 0.944].

Regarding the 3Nb4A-cELISA, the optimal cutoff was 43.61% PI, with a sensitivity of 96.6% and specificity of 81.3%. The 95% CI for the AUC was [0.833, 0.928].

In the case of the 3Nb12H-cELISA, the optimal cutoff was 80.93% PI, with a sensitivity of 92.6% and specificity of 57.7%. The 95% CI for the AUC was [0.688, 0.812].

In summary, 3Nb1A-cELISA demonstrated a more balanced overall performance among the tested assays, with relatively high sensitivity and specificity and a narrow 95% CI for the AUC. These findings indicate that it may be considered for further evaluation.

### 3.7. Analytical Performance of the Developed 3Nb1A-cELISA

The analytical specificity of the 3Nb1A-cELISA was evaluated using *Brucella* antibody-positive serum and sera positive for antibodies against other pathogens. Based on PI values calculated from the OD_450_ readings shown in Figure 5B, the Brucella anti-body-positive serum yielded a PI value ≥ 55.78% and was classified as positive, whereas sera against non-Brucella pathogens showed PI values < 55.78% and were classified as negative, indicating high analytical specificity of the assay.

To assess analytical sensitivity, four *Brucella* antibody-positive sera were serially diluted (1:5, 1:10, 1:20, 1:40, 1:80, and 1:160) to determine the LOD. The assay remained positive at a dilution of 1:20 but became negative at higher dilutions; therefore, the LOD was defined as 1:20 (Figure 5C), indicating good analytical sensitivity of the assay.

Assay repeatability was assessed using three *Brucella* antibody-positive and three *Brucella* antibody-negative sera. The intra-assay CVs ranged from 2.43% to 6.14% (median: 4.12%), and the inter-assay CV ranged from 4.73% to 8.90% (median: 6.53%) (Table 3), indicating good repeatability.

### 3.8. Agreement Analysis of the 3Nb1A-cELISA with SAT and a Commercial ELISA Kit

Using a total of 288 serum samples, the 3Nb1A-cELISA was evaluated in parallel with SAT and a commercial LPS-based ELISA kit to assess agreement. Compared with SAT, the 3Nb1A-cELISA showed a positive percent agreement (PPA) of 73.07%, a negative percent agreement (NPA) of 92.38%, and an overall percent agreement (OPA) of 87.15%, with a κ of 0.668, indicating substantial agreement. In comparison with the commercial ELISA kit, the 3Nb1A-cELISA yielded a PPA of 72.83%, an NPA of 93.23%, and an OPA of 87.50%, with κ = 0.681, also indicating substantial agreement (Table 4). For reference, the commercial ELISA kit showed excellent agreement with SAT (PPA: 97.43%, NPA: 97.61%, OPA: 97.56%; κ = 0.939; McNemar’s test, *p* = 0.453), indicating a high level of agreement. Overall, McNemar’s test showed no significant differences in discordant classifications between the 3Nb1A-cELISA and either SAT or the commercial ELISA (*p* > 0.05).

## 4. Discussion

Brucellosis remains a major zoonotic disease worldwide [6], and serological assays play a central role in its epidemiological surveillance and control. ELISAs based on S-LPS are widely used due to their high sensitivity and standardized protocols. However, structural similarities between LPS O-polysaccharides and those of other Gram-negative bacteria can result in cross-reactivity, thereby compromising specificity [27]. This limitation underscores the need for alternative or complementary diagnostic approaches based on non-LPS antigens. Among these, outer membrane proteins (OMPs) have received increasing attention owing to their immunogenicity and relative species specificity.

OMP16, a conserved outer membrane protein of *Brucella*, has been extensively evaluated as a diagnostic antigen, although its reported performance varies across studies [12,28,29]. While some studies have reported relatively low sensitivity, others have demonstrated improved performance under specific conditions [12]. In the present study, the OMP16-based cELISA achieved a sensitivity of 87.7% and a specificity of 89.4%, indicating moderate diagnostic performance that aligns with previous findings. Several factors may underlie this level of sensitivity. Although OMP16 is conserved among *Brucella* species, it is less immunogenic than LPS. In addition, host antibody responses vary with individual differences and stages of infection [30,31], particularly during early infection when antibody levels are relatively low. Furthermore, owing to their small size and unique antigen-binding structures [32,33], nanobodies may recognize specific or relatively restricted epitopes on OMP16, meaning that detection relies on antibodies targeting specific regions. In the cELISA format used here, antibody detection depends on competition with the probe for these limited epitopes and is therefore influenced by antibody affinity and concentration. Together, these factors likely contribute to the observed limitation in sensitivity.

Despite this, the assay offers several practical advantages. Both the coating antigen (GST-OMP16) and the detection probe (3Nb1A-HRP) are recombinant proteins, eliminating the need for LPS extraction from pathogenic *Brucella* strains and thereby improving biosafety and facilitating standardized production. In addition, the nanobody–HRP fusion enables a secondary antibody-free format, simplifying the assay workflow, reducing dependence on species-specific reagents, and lowering costs. Under the current testing conditions, no significant cross-reactivity was observed with antibodies against several common animal pathogens, suggesting that the assay has favorable specificity. These features support its potential as a complementary non-LPS-based tool for serological detection.

Several limitations should also be considered. From an application standpoint, commonly used vaccines such as A19 and Rev.1 induce antibodies against multiple *Brucella* antigens, including OMP16, meaning that this assay cannot differentiate infected from vaccinated animals (DIVA). This limitation is particularly relevant in the context of disease control and eradication programs. From a study design perspective, all serum samples were obtained from a single geographic region, which may limit the generalizability of the findings. In addition, the scope of pathogens included in the cross-reactivity assessment, as well as the corresponding sample sizes, remains limited and may not fully represent assay performance under more complex field conditions.

Future work may address these limitations through several strategies. Incorporating OMP16 into multi-antigen or multi-epitope diagnostic systems could help improve sensitivity by overcoming the constraints of a single antigen. Previous studies have shown that combining antigens such as Omp25, Omp31, and BP26, or constructing multi-epitope fusion proteins, can enhance sensitivity while maintaining high specificity [34,35]. In parallel, the development of DIVA-compatible assays may be achieved by introducing differential antigens or additional biomarkers. Moreover, further validation using larger sample sets from diverse geographic regions and epidemiological backgrounds will be essential. Expanding the range of heterologous pathogen sera included in validation studies will also allow a more comprehensive assessment of assay specificity, stability, and performance under complex serum conditions.

Overall, this study explored a nanobody-based diagnostic strategy using non-LPS antigens rather than aiming to replace existing LPS-based assays, which are likely to remain the primary tools for brucellosis serological screening. Importantly, the findings demonstrate the feasibility of constructing a competitive ELISA platform based on recombinant antigens and multivalent nanobody probes. This approach provides a practical framework for developing safer and potentially more specific serological assays. With further optimization and broader validation, the platform may serve as a useful complementary tool within the existing diagnostic landscape for brucellosis.

## 5. Conclusions

In this study, we established a recombinant OMP16-based cELISA using a 3Nbs-HRP fusion probe for brucellosis serodiagnosis. The assay exhibited moderate sensitivity and good specificity without detectable cross-reactivity, demonstrating its feasibility as a non-LPS diagnostic approach. By avoiding LPS extraction and incorporating a direct nanobody-HRP fusion format, this strategy enhances biosafety, standardization potential, and cross-species applicability. Although it is not intended to replace conventional LPS-based assays, it provides a complementary diagnostic approach for improving specificity in brucellosis surveillance programs.

## Figures and Tables

**Table 1:** Enrichment of nanobodies against GST-OMP16.

Round of Panning	Input Phage (PFU/Well)	P Output Phage (PFU/Well)	N Output Phage (PFU/Well)	Recovery (P/Input)	P/N *
1st round	5 × 10^10^	2.0 × 10^5^	1.0 × 10^3^	0.4 × 10^−5^	2.0 × 10^2^
2nd round	5 × 10^10^	5.0 × 10^5^	2.0 × 10^2^	1.0 × 10^−5^	2.5 × 10^3^
3rd round	5 × 10^10^	1.5 × 10^6^	1.0 × 10^2^	3.0 × 10^−5^	1.5 × 10^4^

* P/N = Positive output phage (PFU/well)/Negative output phage (PFU/well).

**Table 2:** Cutoff values and diagnostic performance of the three cELISAs.

Assay	Optimal Cutoff (PI)	Sensitivity	Specificity	95% Confidence Interval (CI) for AUC
3Nb1A-cELISA	55.78%	87.70%	89.40%	[0.858, 0.944]
3Nb4A-cELISA	43.61%	96.60%	81.30%	[0.833, 0.928]
3Nb12H-cELISA	80.93%	92.60%	57.70%	[0.688, 0.812]

**Table 3:** Repeatability of the 3Nb1A-cELISA.

Assay	Precision	CV Range (%)	Median CV (%)
3Nb1A-cELISA	Intra-assay	2.43–6.14	4.12
	Inter-assay	4.73–8.90	6.53

**Table 4:** Pairwise diagnostic agreement among the 3Nb1A-cELISA, SAT, and a commercial cELISA kit *.

Assay	Status	3Nb1A-cELISA						
		P	N	PPA	NPA	OPA	κ (Kappa)	McNemar’s Test (*p*-Value)
SAT	P	57	21	73.07%	92.38%	87.15%	0.668	*p* = 0.511
	N	16	194					
Commercial cELISA	P	59	22	72.83%	93.23%	87.5%	0.681	*p* = 0.243
	N	14	193					
Commercial cELISA				97.43%	97.61%	97.56%	0.939	*p* = 0.453
SAT	P	76	2					
	N	5	205					

* P = positive serum; N = negative serum.

## Data Availability

All data generated or analyzed during this study are available from the corresponding author on reasonable request.

## Supplementary Materials

The following supporting information can be downloaded at: https://doi.org/10.6084/m9.figshare.31827424, Table S1: Optimal GST-OMP16 antigen concentration and 3Nb1A-HRP fusion protein; Table S2: Optimal GST-OMP16 antigen concentration and 3Nb4A-HRP fusion protein; Table S3: Optimal GST-OMP16 antigen concentration and 3Nb12H-HRP fusion protein; Table S4: Optimal serum dilution for the OMP16-cELISAs.

## Abbreviations

3Nbs-HRP	Trivalent nanobody–horseradish peroxidase
CV	Coefficient of variation
HRP	Horseradish Peroxidase
iELISA	indirect ELISA
IFA	Immunofluorescence Assay
LOD	Limit of detection
LPS	Lipopolysaccharide
Nbs	Nanobodies
NPA	Negative percent agreement
OMPs	Outer Membrane Proteins
OPA	overall percent agreement
PFU	Phage Forming Unit
PPA	Positive percent agreement
PI	Percentage Inhibition
RBPT	Rose Bengal Plate Test
ROC	Receiver Operating Characteristic
SAT	Standard Tube Agglutination Test
